# Multiple transisthmian divergences, extensive cryptic diversity, occasional long‐distance dispersal, and biogeographic patterns in a marine coastal isopod with an amphi‐American distribution

**DOI:** 10.1002/ece3.2397

**Published:** 2016-10-06

**Authors:** Luis A. Hurtado, Mariana Mateos, Gustavo Mattos, Shuang Liu, Pilar A. Haye, Paulo C. Paiva

**Affiliations:** ^1^ Department of Wildlife and Fisheries Sciences Texas A&M University College Station Texas; ^2^ Programa de Pós‐Graduação em Ecologia Universidade Federal do Rio de Janeiro Rio de Janeiro Brazil; ^3^ Departamento de Biología Marina Universidad Católica del Norte Coquimbo Chile; ^4^ Centro de Estudios Avanzados en Zonas Áridas (CEAZA) Coquimbo Chile; ^5^ Interdisciplinary Center for Aquaculture Research (INCAR) Universidad de Concepción Casilla 160‐C Concepción Chile; ^6^ Departamento de Zoologia Universidade Federal do Rio de Janeiro Rio de Janeiro Brazil

**Keywords:** Cirolanidae, cryptic diversity, dispersal, historical biogeography, Panama Isthmus, vicariance

## Abstract

*Excirolana braziliensis* is a coastal intertidal isopod with a broad distribution spanning the Atlantic and Pacific tropical and temperate coasts of the American continent. Two separate regional studies (one in Panama and one in Chile) revealed the presence of highly genetically divergent lineages, implying that this taxon constitutes a cryptic species complex. The relationships among the lineages found in these two different regions and in the rest of the distribution, however, remain unknown. To better understand the phylogeographic patterns of *E. braziliensis*, we conducted phylogenetic analyses of specimens from much of its entire range. We obtained DNA sequences for fragments of four mitochondrial genes (16S rDNA, 12S rDNA, COI, and Cytb) and also used publicly available sequences. We conducted maximum likelihood and Bayesian phylogenetic reconstruction methods. Phylogeographic patterns revealed the following: (1) new highly divergent lineages of *E. braziliensis*; (2) three instances of Atlantic–Pacific divergences, some of which appear to predate the closure of the Isthmus of Panama; (3) the distributional limit of highly divergent lineages found in Brazil coincides with the boundary between two major marine coastal provinces; (4) evidence of recent long‐distance dispersal in the Caribbean; and (5) populations in the Gulf of California have closer affinities with lineages further south in the Pacific, which contrasts with the closer affinity with the Caribbean reported for other intertidal organisms. The high levels of cryptic diversity detected also bring about challenges for the conservation of this isopod and its fragile environment, the sandy shores. Our findings underscore the importance of comprehensive geographic sampling for phylogeographic and taxonomical studies of broadly distributed putative species harboring extensive cryptic diversity.

## Introduction

Documentation of cryptic species (i.e., those that are difficult or impossible to distinguish on the basis of morphology) has increased exponentially in the last couple of decades and is associated with the widespread application of DNA sequencing (Bickford et al. 2007). Cryptic diversity can bring about serious taxonomic challenges and severely limit or mislead inferences on evolution, ecology and conservation status of affected taxa (Beheregaray and Caccone 2007; Bickford et al. 2007; Pfenninger and Schwenk 2007; Trontelj and Fiser 2009). Although cryptic diversity occurs in many taxonomic groups, geographic regions and habitats, whether or not it is evenly distributed among these is under debate (Pfenninger and Schwenk 2007; Trontelj and Fiser 2009). Notwithstanding, certain kinds of metazoans appear especially prone to harboring high levels of cryptic diversity, including subterranean fauna (e.g., Trontelj et al. 2009; Zaksek et al. 2009; Niemiller et al. 2013; Zhang and Li 2014), and isopods that inhabit the intertidal zone (references below). Poore and Bruce (2012) estimate that approximately four‐fifths of intertidal isopod species remain unknown.

Biological characteristics of intertidal isopods, such as direct development (a feature of all peracarids) and habitat specificity, appear to severely limit their dispersal potential, which can result in high levels of isolation and genetic differentiation among populations. Concordantly, recent phylogeographic studies of intertidal isopods indicate that several species presumed to occupy broad distributions, instead correspond to diverse species complexes comprised of multiple highly divergent cryptic lineages, which often have very restricted geographic ranges (Sponer and Lessios 2009; Hurtado et al. 2010, 2013, 2014; Varela and Haye 2012; Santamaria et al. 2013, 2014, 2016). Their phylogeographic patterns are of biogeographic relevance because they retain signatures consistent with past geological and climatic events, environmental factors, as well as instances of overwater dispersal. Given the broad distribution of currently valid species of several coastal isopods, the extensive cryptic diversity they tend to harbor, and the restricted ranges of cryptic lineages, accurate inferences regarding their phylogeography, evolution, and diversity require comprehensive sampling throughout their distributional ranges.

**Figure 1:**
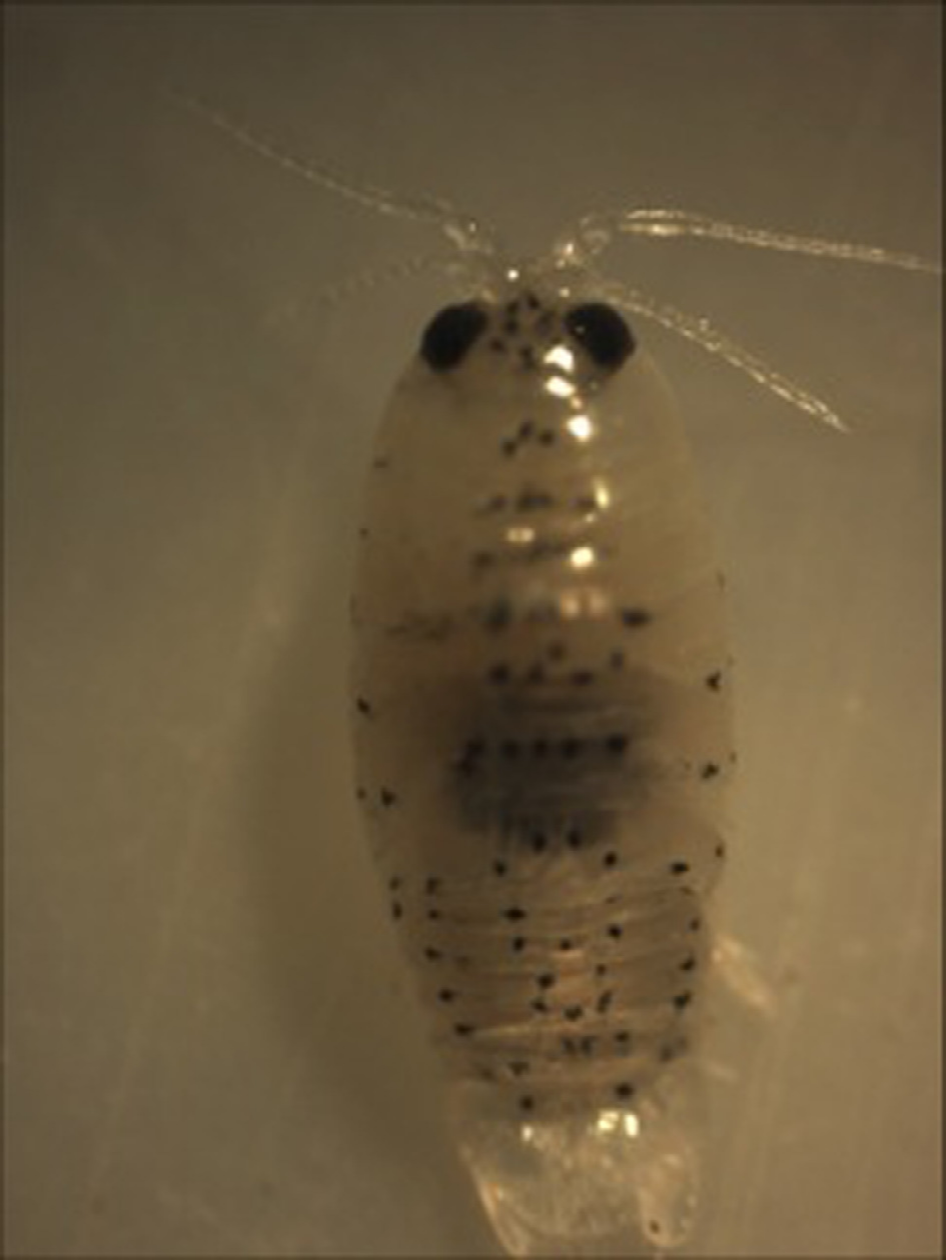
Photograph of a specimen of *Excirolana braziliensis*.

**Figure 2:**
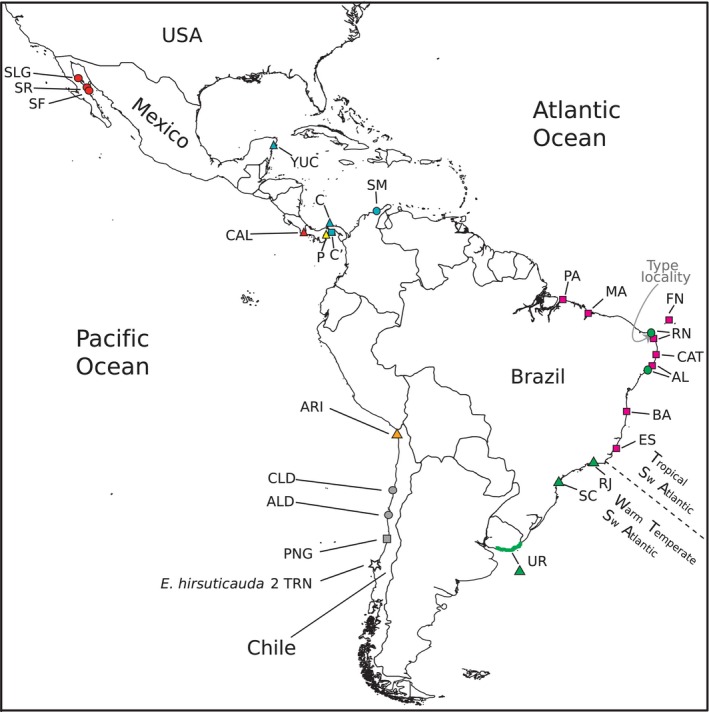
Sampled localities for *Excirolana braziliensis*. Color‐coding and locality abbreviation correspond with other figures and tables. Detailed information for each locality is presented in Table S1. The locality of the outgroup taxon *Excirolana hirsuticauda* is indicated by a star. A dashed line indicates the boundary between the tropical southwestern (SW) Atlantic and the warm temperate southwestern (SW) Atlantic marine provinces. Base map created by C. Smith in ArcGIS V.10.3 (Redlands, CA: Esri, 2014); Administrative Units (admin.shp), Edition 10.1, ArcWorld Supplement, 2012.

The coastal isopod *Excirolana braziliensis* Richardson, 1912 (Fig. 1) is a nominal species presumed to have a broad amphi‐American distribution, extending along the Pacific coast from the Gulf of California to southern Chile, and along the Atlantic coast from the Gulf of Mexico to Uruguay, including the Caribbean (Glynn et al. 1975; Brusca et al. 1995). Regarded as one of the most abundant macroscopic animals inhabiting the sandy beaches of this region (Glynn et al. 1975), this isopod has been the subject of important research on ecology of sandy beaches (Glynn et al. 1975; Dexter 1976, 1977; Fonseca et al. 2000; Cardoso and Defeo 2003, 2004; Defeo and Martínez 2003; Martínez and Defeo 2006) and marine connectivity (e.g., Weinberg and Starczak 1988, 1989; Lessios and Weinberg 1993, 1994; Lessios et al. 1994; Sponer and Lessios 2009; Varela and Haye 2012; Tourinho et al. 2016). It was described from a single specimen collected off Cabo São Roque, Brazil (Richardson 1912), in the easternmost region of the American continent (Fig. 2). A taxonomic revision based on specimens from 21 localities along the eastern Pacific from Mexico to Chile and 20 localities in the western Atlantic from Mexico to Brazil, including the West Indies, concluded that *E. braziliensis* constitutes a single valid species (Glynn et al. 1975). Accordingly, *Cirolana salvadorensis* Schuster and *Cirolana koepckei* Bott correspond to different growth forms of *E. braziliensis*, and are thus, junior synonyms (Glynn et al. 1975). These authors note, however, that the sporadic presence of very large adults with extreme morphological modifications in Pacific, but not Atlantic, populations is puzzling and contributes to the taxonomic confusion surrounding this species. A subsequent taxonomic revision (Brusca et al. 1995) also concluded that *E. braziliensis* constitutes a single species with a broad amphi‐American distribution that spans tropical, subtropical and temperate beaches. Its range largely overlaps with that of *Excirolana mayana* Ives, in the Atlantic and Pacific, and that of *Excirolana chamensis* Brusca and Weinberg, a highly restricted species reported in the Pacific coast of Panama (Brusca and Weinberg 1987; Brusca et al. 1995). These three species, however, have been confused in the ecological literature (Brusca et al. 1995).

Genetic studies conducted in three separate regions revealed that *E. braziliensis* likely consists of multiple apparently cryptic species. In Panama, allozyme, mitochondrial (i.e., fragments of the 12S ribosomal(r)RNA and the cytochrome oxidase I [COI] genes) and morphological data indicate the presence of three highly divergent lineages suggested to represent three separate species: one from the Caribbean (C morph) and two from the Pacific (C′ and P morphs) (Sponer and Lessios 2009). Of these, the C and C′ morphs constitute reciprocally monophyletic sister lineages, whose divergence is placed at ~6–17 Mya (Sponer and Lessios 2009), thereby predating the final closure of the Isthmus of Panama (~3 Mya). These authors also suggest that the divergence between the ancestor of the C and C′ lineages and the P morph lineage occurred ~9–25 Mya. A separate molecular study (based on a largely nonoverlapping portion of the COI gene; Varela and Haye 2012) found also three highly divergent allopatric lineages of *E. braziliensis* in the coast of Chile (Pacific), each of which was suggested to represent a distinct species. According to their distribution, these Chilean lineages were designated as follows: northern clade, center clade, and southern clade (hereafter referred to as “N,” “M,” and “S” clades, respectively). The latter two were found to be reciprocally monophyletic and sister to the northern clade. A third molecular study revealed that *E. braziliensis* from Uruguay represents a highly divergent lineage to the ones found in Chile and Panama (Tourinho et al. 2016). Phylogenetic relationships among the lineages found in these regions have not been established.

The cryptic diversity reported to date within *E. braziliensis* appears consistent with the low dispersal potential of this isopod. According to Glynn et al. (1975), adults of *E. braziliensis* are confined to the highest tidal level, where they burrow into sand, whereas juveniles are most common at lower reaches of the beach. Morphological and molecular studies of the three morphs in Panama indicate that gene flow between populations located <5 km apart can be highly restricted (Lessios and Weinberg 1993) and populations >30 km apart are genetically divergent (Sponer and Lessios 2009). Occasional dispersal and rare extinction–recolonization events, however, have been documented in the Pacific coast of Panama (Lessios et al. 1994).

To achieve a comprehensive understanding of the genetic diversity and evolutionary history of *E. braziliensis*, it is crucial to obtain a better representation from its entire distribution. Herein, we conducted phylogenetic analyses of specimens from much of its geographic range. Due to its potential for harboring high levels of cryptic diversity, we expected to uncover additional hidden diversity within *E. braziliensis*. In addition, in light of the wide distribution and apparently long evolutionary history of this isopod, three biogeographic questions were addressed. First, as this isopod has an amphi‐American distribution, it is important to understand the patterns of divergence of Atlantic and Pacific lineages. The complex Miocene–Pliocene geological history associated with the emergence of the Isthmus of Panama may have provided multiple opportunities for Atlantic–Pacific vicariance of marine taxa (Bacon et al. 2015), which could have left signatures in a coastal organism with the biological characteristics of *E. braziliensis*. Second, the broad distribution of this isopod spans multiple biogeographic regions (e.g., provinces and ecoregions), whose boundaries may constitute barriers for dispersal, as has been observed in other coastal isopods (e.g., Eberl et al. 2013). Our sampling in Brazil encompasses the boundary between the tropical southwestern Atlantic and the warm temperate southwestern Atlantic marine provinces (Spalding et al. 2007), providing the opportunity to examine whether the geographic limits of lineages correspond to this division. Third, we also examined whether populations of this isopod in the Gulf of California have closer affinities with lineages from the Caribbean, as has been observed for other coastal isopods (Hurtado et al. 2013, 2014) and for marine fossils (Smith 1991; Escalona‐Alcázar et al. 2001).

## Materials and Methods

### Samples

We examined newly generated (*n* = 121) and published (i.e., 11 from Chile, 105 from Panama, and 13 from Uruguay) sequences of *E. braziliensis* from localities in the Pacific and Atlantic coasts of America representing the distributional range of *E. braziliensis* (Fig. 2; Table S1). Detailed new sampling effort focused on two regions: the Gulf of California, where only three localities (out of >50 surveyed) had *E. braziliensis*, and the Brazilian coast (12 localities). The latter spanned three marine provinces (sensu Spalding et al. 2007): the North Brazil Shelf (Bragança, Pará [PA], and São Marcos Beach, São Luis, Maranhão [MA]); the tropical southwestern Atlantic (from Sueste Beach, Fernando de Noronha Archipelago [FN] to Guarapari, Espírito Santo [ES]); and the warm temperate southwestern Atlantic (Rio de Janeiro [RJ] and Florianópolis, Santa Catarina [SC]). One Pacific (Costa Rica) and two Caribbean (Mexico and Colombia) localities were also included. For Chile, we generated new sequences from representative specimens of the main clades found in the study of Varela and Haye (2012). Details on how published and new sequences, which had partial overlap in genes or gene fragments, were combined are given below and in Table S1. For tree rooting purposes, we used samples of *E. mayana* from the Gulf of California and the Caribbean (Hurtado, unpublished data), *Excirolana hirsuticauda* from Chile, and *Excirolana chiltoni* from Korea and Oregon.

Specimens were assigned to *E. braziliensis* on the basis of diagnostic morphological traits examined under a dissecting microscope, namely a conspicuous transverse depression on the telson and long antennules extending to pereonite IV (Richardson 1912; Glynn et al. 1975; Brusca et al. 1995). Although this isopod is similar to *E. chamensis*, it can be distinguished by the three‐articulate mandibular palp (which is two articulate in *E. chamensis*) and a not‐fully‐divided endopod on pleopod V (which is fully divided into *E. chamensis*) (Brusca and Weinberg 1987).

### Molecular methods

Genomic DNA was extracted from leg tissue with the DNEasy kit (Qiagen, Inc., Valencia, CA). Four mitochondrial gene fragments were amplified as follows: a ~439‐bp fragment of the 16S rDNA gene and a ~480‐bp fragment of the 12S rDNA gene using the primers reported in Podsiadlowski and Bartolomaeus (2005); a 430‐bp fragment of the cytochrome b gene (Cytb) using the primers reported in Merritt et al. (1998); and a 710‐bp fragment of the COI gene using the primers reported in Folmer et al. (1994). PCR‐amplified products were cleaned with exonuclease and shrimp alkaline phosphatase and sequenced at the University of Arizona Genetics Core. We used Sequencher 4.8 (Gene Codes, Ann Arbor, MI) for sequence editing and primer removal. For a subset of localities, we obtained Cytb sequences from 3 to 5 individuals to examine genetic variation within localities. As most localities exhibited little variation among individuals (Fig. S1), only one individual was further characterized for the additional genes.

### Datasets and sequence alignments

Because for some taxa only sequences from one gene were publicly available or obtained in our study, we conducted phylogenetic analyses on two kinds of datasets: a concatenated dataset of the four mitochondrial genes including only specimens containing all four genes and datasets of individual genes (12S rDNA and 16S rDNA). The 12S rDNA dataset was the only one that included the samples from Panama (i.e., 105 sequences representing the C, C′, and P morphs) reported in Sponer and Lessios (2009). Although Sponer and Lessios (2009) also generated COI sequences, the region of overlap with this and previous *E. braziliensis* studies is too short to be of phylogenetic utility here and thus not presented. For some Brazilian localities, we were unable to obtain 12S rDNA, COI, or Cytb sequences, but 16S rDNA sequences were obtained for all Brazilian localities. For the Uruguay localities, only sequences from COI (*n* = 13) were available (Tourinho et al. 2016).

For each dataset, we conducted a separate alignment. Sequence alignments were performed with the online version of MAFFT v.6.0 (Katoh and Toh 2008). Because there was little difference between the alignments obtained from the L‐INS‐I strategy and Q‐INS‐I strategy, we chose the former one. For protein‐coding genes, we used MacClade v.4.08 (Maddison and Maddison 2005) to check for premature stop codons. We used the online version of GBlocks v.0.91b (Castresana 2000) to identify positions that should be excluded due to questionable homology. The following parameters were assumed: “Allowed Gap Positions” = half; “Minimum Length of A Block” = 0.5 or 10; and “Maximum Number of Contiguous Nonconserved Positions” = 4 or 8 for removing uncertain homology sequence blocks.

### Phylogenetic analyses

We used the program jModeltest v0.1.1 (Posada 2008) to select the most appropriate DNA substitution model among 88 candidate models computed from a fixed BioNJ‐JC tree. We used the following three criteria: the Akaike information criterion (AIC), corrected AIC(c), and Bayesian information criterion (BIC) (Table S2). We conducted maximum likelihood (ML) and Bayesian analyses. ML analyses were performed in RaxMLGUI v. 1.0, which implements the Rapid Bootstrap followed by ML search of RAxML 7.3.0 (Stamatakis 2006; Silvestro and Michalak 2012). We also used GARLI v.2.0 (Zwickl 2006) with bootstrap searches, which employs genetic algorithms for the ML search. Bootstrap trees were summarized (50% majority rule consensus) with the SumTrees script of DendroPy v.3.10.1 (Sukumaran and Holder 2010). Bayesian analyses were performed within MrBayes v.3.2.1 (Huelsenbeck et al. 2001; Ronquist and Huelsenbeck 2003; Ronquist et al. 2012) and Phycas v.1.2.0 (Lewis et al. 2010), implementing the polytomy prior (Lewis et al. 2005) in the latter. The polytomy prior is one of the proposed ways to address problems arising from the “star‐tree paradox” (Lewis et al. 2005). For the MrBayes analyses, we used 10 billion generations and collected a tree every 1000 generations. In both, ML and Bayesian analyses, we used the closest more complex model available if the best models identified by jModeltest could not be implemented (Table S3). Otherwise, when the combination of a proportion of invariable sites (I) and a Gamma distribution of rates among sites (G) was selected as the best model, the parameter (I) was removed to avoid the potential problems related to dependency between two parameters (see RaxML manual and Yang 2006). In addition to the single‐partition analyses described above, datasets of concatenated genes were also analyzed under a multiple partition approach. The most appropriate partitioning scheme was identified with PartitionFinder v.1.0 (Lanfear et al. 2012). For noncoding genes, each gene was assigned to a different partition, whereas for coding genes, each gene and codon position (i.e., first, second, and third) was assigned to a separate partition. The following parameters were used in PartitionFinder: branch lengths = linked; models = all; model selection = BIC; search = greedy. The selected partitioning scheme is shown in Table S3.

Convergence and adequate sampling of the posterior distribution was assessed by (1) stable posterior probability values in more than 200 samples (Effective Sample Size) in Tracer v.1.5 (Rambaut and Drummond 2007); (2) a high correlation between the split frequencies of independent runs as implemented in AWTY (Nylander et al. 2008); (3) small and stable average standard deviation of the split frequencies of independent runs; and (4) Potential Scale Reduction Factor close to 1. Samples prior to reaching a stationary posterior distribution were eliminated as burn‐in (Table S3).

Pairwise genetic distances were estimated for the COI, 16S rDNA, 12S rDNA genes separately with the Kimura‐2‐parameter (K2P) correction implemented in MEGA v.5 (Tamura et al. 2011) or PAUP* v. 4.0b10 (Swofford 2002). Pairwise genetic distances were estimated on datasets that excluded positions of questionable homology and using pairwise deletion for missing data.

## Results

**Figure 3:**
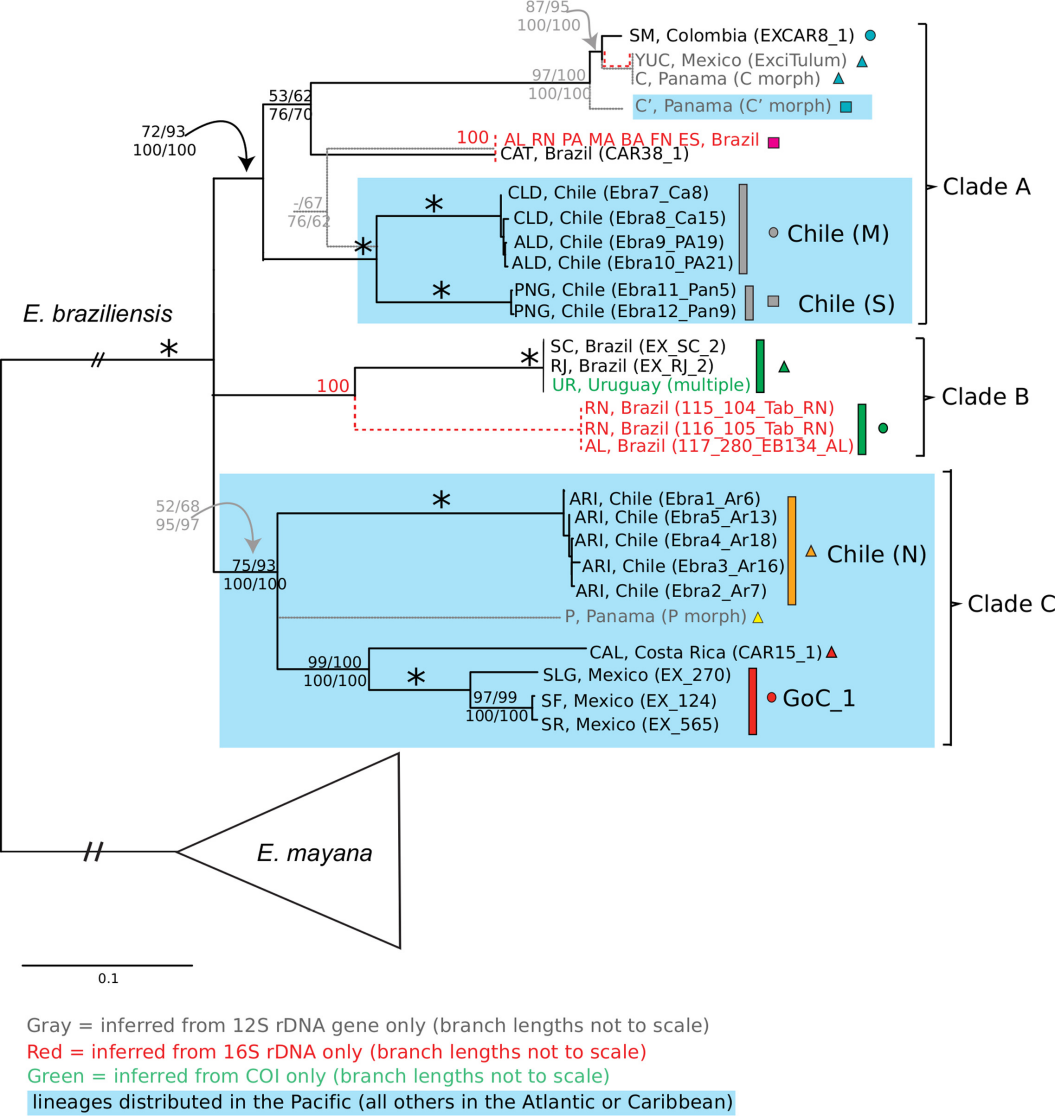
Inferred phylogeny of *Excirolana braziliensis* based on four concatenated mitochondrial genes. RaxML bootstrap majority rule consensus tree inferred with the Dataset S5 of four concatenated mitochondrial genes (12S + 16S + Cytb + COI) and including *Excirolana mayana* as the only outgroup. Lineages indicated by red font and branches were redrawn on the basis of the 16S rDNA dataset and those indicated by gray font and branches were redrawn on the basis of the 12S rDNA dataset. Numbers by nodes indicate the corresponding Bootstrap Support (BS; top or left) for maximum likelihood (RaxML and Garli, respectively) and posterior probabilities (PP; bottom or right) for Bayesian inference methods (MrBayes and Phycas, respectively), including all partitioning schemes. * denotes nodes that received 100% support for all methods. – denotes nodes receiving <50% support for the corresponding method. Nodes receiving <50% support for all methods were collapsed. Colors and shapes correspond to lineages, clades or localities in other figures. Blue shading indicates taxa found in the Pacific Ocean.

Phylogenetic reconstructions indicate the presence of highly genetically divergent cryptic lineages of *E. braziliensis*. Figure S2 shows the phylogenetic tree of the 12S rDNA dataset, which is the only one that contains the samples reported in Sponer and Lessios (2009), corresponding to the morphs identified as C (Caribbean Panama), C′ (Pacific Panama), and P (Pacific Panama). Figure S3 shows the phylogenetic reconstruction of the 16S rDNA dataset, which is the only dataset that contains all of the Brazilian localities. Figures 3 and S4 show the phylogenetic tree of the four concatenated mitochondrial genes dataset. We superimposed the phylogenetic position of the Panama Brazil and Uruguay specimens, for which only the 12S rDNA, 16S rDNA, and COI sequences, respectively, were available. The tree in Fig. S4, which was rooted with *E. hirsuticauda* and *E. chiltoni*, revealed the reciprocal monophyly of *E. braziliensis* and *E. mayana*. The tree in Fig. 3 was inferred without *E. hirsuticauda* and *E. chiltoni* and, thus, was rooted at the branch joining *E. braziliensis* and *E. mayana*. Relationships within *E. braziliensis* inferred by the two rooting strategies were mostly concordant. Genetic distances are shown for the 12S rDNA, 16S rDNA, and COI datasets (Tables S8–S10). All new sequences produced in this study have been deposited in GenBank under accession Numbers KT122410–KT122766, KX530933–KX530936, KX530938–KX530940 (Table S1). Annotated alignments used in analyses are in Datasets S1–S5.

Three main lineages joined at a basal trichotomy, which differ from each other at 7.2–17% for the 12S rDNA gene (K2P; Table S8) and 18–25% at COI (Table S10), were recovered for *E. braziliensis* (Figs. 3, S4). The first main lineage (Clade A) is comprised of three clades (maximum 12S rDNA K2P divergence among them = 11.4%; Table S6): (1) a clade that contains the samples of the C′ morph from Panama's Pacific coast (turquoise square; Figs. 2, 3, S2, and S4), which is sister to a group formed by the samples of the C morph from Panama's and Mexico's Caribbean coast (turquoise triangle; 12S rDNA sequences from Mexico's Caribbean coast were identical to some of the C morph sequences from Panama) and from Santa Marta, Colombia (turquoise circle); (2) a clade comprised of tropical Brazilian samples (magenta square) from multiple localities [we obtained sequences from all mitochondrial genes for the samples from Catuama, Pernambuco (CAT); for the other localities, which were not highly divergent from CAT, we obtained only 16S rDNA sequences; see Fig. S3]; and (3) a clade that includes the samples from southern Chile (gray square), which is sister to the samples from central Chile (Clade “Chile M;” gray circle; 3.9% divergent from each other at 12S rDNA; and ~16% at COI). The second main lineage (Clade B) was exclusively found in Brazil and Uruguay (green) and included a clade made up of the warm temperate southwestern Atlantic samples (green triangle) of Rio de Janeiro (RJ), Santa Catarina (SC) and the eight localities from Uruguay (maximum COI K2P within‐clade divergence = 0.64%; Table S10), which is sister to a highly divergent lineage (11.1–11.9% at 16S rDNA; Table S9) found further north (green circle) at Rio Grande do Norte (RN) and Alagoas (AL). Thus, two divergent lineages (i.e., from clades A and B) co‐occur at these latter two localities (only 16S rDNA sequences were obtained). As the type locality of *E. braziliensis* (i.e., off Cabo São Roque, Brazil; Fig. 2) is located ~ 110 km south of the RN locality, there is uncertainty concerning the lineage to which the holotype of *E. braziliensis* belongs. The third main lineage (Clade C) was exclusively found in the Pacific and is comprised of the following three lineages joined at a basal trichotomy (maximum divergence = 13.4, 13.5, and 21% at 12S rDNA, 16S rDNA, and COI, respectively): (1) a lineage with the samples of northern Chile (orange triangle); (2) a lineage with the samples of the P morph (Pacific Panama; yellow triangle); and (3) a clade made up of the samples from Pacific Costa Rica (red triangle), which is sister to (and 7.7–8.5% divergent at 12S rDNA from) the clade comprised of the three localities sampled in the Gulf of California (red circle).

**Figure 4:**
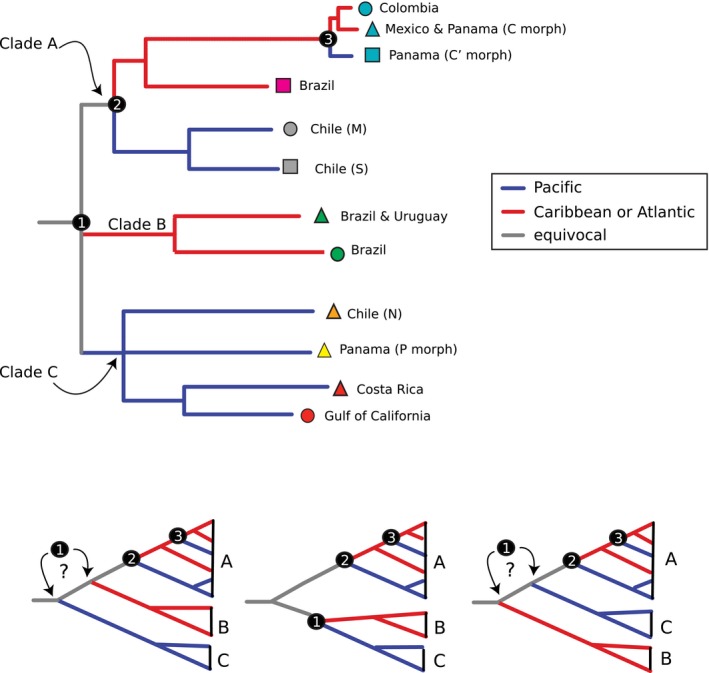
Summarized phylogeographic patterns. Clade labels and locality symbols/colors correspond to those in other figures. Numbered nodes represent the inferred Atlantic–Pacific divergence events. Branch colors indicate most parsimonious location of lineage (i.e., Pacific, Atlantic, equivocal). Branches are not drawn to scale. Bottom panel depicts alternative scenarios for Atlantic–Pacific divergence number 1, depending on alternative solutions to unresolved relationships.

Signatures consistent with three Atlantic–Pacific divergences are observed in the phylogeographic patterns of *E. braziliensis*. Based on the order of divergences and genetic distances, the split between the C morph (Caribbean coast of Panama, Colombia, and Yucatan Peninsula) and the C′ morph (Pacific Panama) in Clade A (4.8–7.3% at 12S rDNA) likely represents the most recent Atlantic–Pacific split (node “3” in Fig. 4). A relatively older Atlantic–Pacific divergence is inferred at the split of the lineage grouping the central (M) and southern (S) Chile clades versus the remaining lineages in Clade A (node “2” in Fig. 4; 5.1–11.4% at 12S rDNA and 11.9–13.6% at 16S rDNA). The third Atlantic–Pacific divergence (node “1” in Fig. 4), possibly older, involves the split of the basal Clade C, restricted to the Pacific, from one or both of the other main clades of *E. braziliensis* (A and B). Divergences at 12S rDNA are as follows: B versus C = 13.5–15.5% and A versus C = 7.2–17.0%.

## Discussion

Two taxonomic revisions (published 20 years apart) based on traditional morphology, which included a comprehensive representation of its entire range, concluded that *E. braziliensis* constitutes a single species (Glynn et al. 1975; Brusca et al. 1995). Subsequent phylogeographic studies of this isopod conducted separately in the coasts of Panama and Chile, however, found that three highly divergent lineages were present in each region (Sponer and Lessios 2009; Varela and Haye 2012). A more recent study revealed that the coast of Uruguay harbors a lineage of *E. braziliensis* that is highly divergent from those in Panama and Chile (Tourinho et al. 2016). Nonetheless, the relationships among lineages of these three regions remained largely unresolved. Our results confirm the finding by Sponer and Lessios (2009) that the C morph (Caribbean) and C′ morph (Pacific) lineages from Panama represent sister groups. Nevertheless, the divergent P morph (Pacific) lineage from Panama is not their closest relative. Similarly, our results confirm the sister relationship reported by Varela and Haye (2012) between the center (M) and southern (S) clades from Chile, but reject their sister relationship to the northern clade from Chile. Instead, our results indicate that the P morph lineage from Panama and the north Chile lineage belong to a divergent clade that also includes previously unknown lineages from the Pacific coast of Costa Rica and the Gulf of California. We identified additional highly divergent lineages of *E. braziliensis* that were not observed in previous studies and improved inferences on the phylogenetic relationships among the presently known lineages of this isopod. Our results illustrate the importance of comprehensive sampling in phylogeographic studies of broadly distributed putative species with extensive cryptic diversity.

A surprising finding of this study was the identification of identical 12S rDNA sequences in *E. braziliensis* specimens from Panama (C morph) and Mexico's Caribbean coast, which suggests recent exchange of individuals between these two regions separated by >1400 km. Although previous observations suggested occasional dispersal for this isopod, it appeared to occur at a much smaller scale. Long‐term sampling of *E.  braziliensis* in Panama indicates some instances of extirpation of local populations and subsequent replacement by new immigrants from nearby localities (Lessios et al. 1994). In addition, a small proportion of immigrants have been detected in Panamanian localities with established populations of *E. braziliensis* (Weinberg and Starczak 1988). Genetic analyses, however, indicate that although some movement of individuals among Panamanian localities might occur, gene flow among different lineages or genotypes is not detectable, suggesting some degree of reproductive isolation (Lessios and Weinberg 1993). Gene flow among Panamanian populations located <5 km apart was shown to be highly restricted (Lessios and Weinberg 1993), whereas populations >30 km apart are genetically divergent (Sponer and Lessios 2009). Sampling between Panama and the Yucatan peninsula will be required to determine whether or not the shared haplotype (and/or its close relatives) occurs throughout the region. *E. braziliensis* is an active swimmer that spends part of its life underwater in the intertidal zone (Jaramillo and Fuentealba 1993) and feeds on animal carcasses, a trait that may facilitate passive long‐distance movements. Nonetheless, vulnerability to fish predation might severely limit the dispersal potential of this isopod (Sponer and Lessios 2009). Hurricanes and/or ballast water may have facilitated the movement of individuals between Panama and the Yucatan peninsula. Long‐distance human‐mediated dispersal, possibly via water ballast, has been suggested in other cirolanids (Bowman et al. 1981), although this has not been assessed at the molecular level.

The high genetic divergences observed among many of the lineages suggest that the isopod *E. braziliensis* corresponds to a complex of cryptic species. Sponer and Lessios (2009) indicate that based on morphological, allozyme, and mitochondrial data, each of the three lineages found in Panama (i.e., the C morph from the Caribbean, the C′ morph from the Pacific, and the P′ morph from the Pacific) represents a distinct species. Similarly, based on the COI marker, Varela and Haye (2012) suggest that the three divergent lineages found in Chile (i.e., northern Chile clade, center Chile clade, and southern Chile clade) also represent distinct species. Given their high genetic divergences, it is likely that the three most divergent lineages from Brazil (one within Clade A and two within Clade B), the lineage from Costa Rica (Pacific coast), and the one from the Gulf of California, both from Clade C, also represent different species. Smaller genetic divergences may also represent different species, such as those observed in the comparison of San Luis Gonzaga versus San Rafael/San Francisquito (1.5, 4, and 9.2% at 12S rDNA, 16S rDNA, and COI, respectively), in the Gulf of California, and also among some Brazilian localities. Similarly, given its divergence, the sample from Colombia (SM) might represent a distinct species from the C morph in Panama and Mexico's Caribbean coast clade (1.2% at 12S rDNA; 3.75% at 16S rDNA). It is possible that additional sampling across the geographic range of *E. braziliensis* will reveal additional divergent lineages or further structure within the lineages identified to date. Other kinds of data, however, are needed (e.g., morphological, nuclear DNA, experimental crossings) to define which lineages constitute different species.

In light of the high cryptic diversity observed, a taxonomic revision of *E. braziliensis* is needed. Previous comprehensive taxonomic revisions based on traditional morphology, however, have failed to recognize cryptic diversity within this isopod (Glynn et al. 1975; Brusca et al. 1995). In the description of *E. chamensis*, Brusca and Weinberg (1987) mentioned that in a forthcoming paper by Brusca, several additional new species of *Excirolana* would be described from the tropical eastern Pacific, and the status of *E. braziliensis* would be reassessed. Nonetheless, in their revision of the Cirolanidae of the tropical eastern Pacific, Brusca et al. (1995) only recognize *E. braziliensis*,* E. mayana*, and *E. chamensis* and concur with the synonymization of *C. salvadorensis* and *C. koepckei* with *E. braziliensis*. The morphometric analyses of Lessios and Weinberg (1994), based on eight characters (body length, body width, eye diameter, rostrum width, length of flagellum on antennule, length of flagellum on antenna, length of last peduncular article on antenna, and length of uropodal exopod), distinguished the C, C′, and P morphs from Panama. An earlier morphometrics study (Weinberg and Starczak 1989), however, which examined the same characters and included samples from throughout the range of *E. braziliensis*, distinguished only two groups: Group I (which contained 14 Pacific and 2 south Atlantic populations) and Group II (which contained 12 Pacific and 17 Caribbean populations). Based on the distribution of these groups, it appears that Group I corresponds to clades B and C in our study, whereas Group II corresponds to our Clade A. Thus, these tools may be useful for discriminating clades of *E. braziliensis* at different levels of divergence. Body shape geometrics–morphometrics represents an alternative approach to examine the extent of cryptic diversity in this isopod. A recent such study of highly divergent lineages of a putative species of the intertidal isopod *Ligia*, however, revealed highly constrained evolution in body shape (Santamaria et al. 2016).

The assumption of a single widespread species has led to misleading inferences regarding the underlying causes of ecological variation. For example, Cardoso and Defeo (2004) examined life‐history traits for *E. braziliensis* from 12 localities along broad latitudinal gradients on both the Atlantic (~23–34°S) and Pacific (~9°N–39°S) coasts. They reported a latitudinal effect (replicated on either coast) on several life‐history traits, including individual size, growth rates and seasonality, life span, and length–mass relationship. On the assumption that such variation occurred within a single species, the authors concluded that phenotypic plasticity was the underlying cause of the observed patterns. The cryptic diversity and phylogeographic patterns of *E. braziliensis* suggest, however, that convergence rather than phenotypic plasticity is responsible for the observed similarities in latitudinal population dynamics. This observation illustrates the importance of accounting for potential cryptic diversity in macroecological studies of taxa with low dispersal potential.

The phylogeographic patterns of *E. braziliensis* contain three signatures congruent with Atlantic–Pacific divergences: two within Clade A and one involving Clade C versus clades A and/or B. Due to unknown substitution rates, lack of appropriate clock calibration points, and the poor resolution at the base of the tree and at the bases of clades A and C, we can only make highly speculative inferences regarding the possible timing of such events on the basis of the order of divergences and genetic distances. Accordingly, the divergence between morph C (Atlantic coast of Panama, Colombia and Mexico) and morph C′ (Pacific Panama) likely occurred more recently than the other two Atlantic–Pacific divergences. On the basis of substitution rates borrowed from other crustaceans, Sponer and Lessios (2009) argue that the C versus C′ split probably predates the final closure of the Isthmus of Panama (~3 Mya) and suggest it occurred 6–17 Mya. Transisthmian divergences predating the final closure of the Isthmus of Panama appear to have been rather common. Lessios (2008) examined molecular divergence data from 115 pairs of transisthmian geminate clades, including crustaceans, sea urchins, fishes, and mollusks, and suggests that ~70 pairs diverged before the Isthmus completion. Nonetheless, without reliable calibration points and/or substitution rates, the final closure of the Isthmus of Panama cannot be completely ruled out as the vicariant event responsible for the C versus C′ divergence. According to the phylogeographic patterns and relative genetic divergences, even if the most recent Atlantic–Pacific divergence (C vs. C′) represents the final Isthmus closure, the other two appear to predate this event. Thus, the likely origin of the *E. braziliensis* clade occurred within the Miocene (~5–23 Mya) or earlier. Sponer and Lessios (2009) estimated the divergence between the ancestor of the C and C′ morph and the P morph lineages occurred ~9–25 Mya, albeit based on borrowed rates that might be inadequate.

A complex geological history associated with the emergence of the Isthmus of Panama appears to have provided multiple opportunities for Atlantic–Pacific vicariance of marine organisms. A recent analysis of a large dataset of molecular phylogenetic studies from this region suggests the occurrence of three separate vicariant events responsible for Atlantic–Pacific divergences in marine organisms at 23.73 (19.9–27.41), 7.96 (7.75–8.96), and 2.06 (1.03–4.35) Mya (Bacon et al. 2015). Separate vicariant events may explain the independent Atlantic–Pacific divergences observed in *E. braziliensis*. Similarly, phylogeographic patterns of the supralittoral isopod *Ligia* revealed two Atlantic–Pacific divergences (Santamaria et al. 2014), which may also reflect separate vicariant events.

Marine biogeographic boundaries correspond to distribution limits of several *E. braziliensis* lineages. In the Brazilian coast, the boundary between the tropical southwestern Atlantic and the warm temperate southwestern Atlantic marine provinces (Spalding et al. 2007) appears to constitute the limit of the most divergent lineages found in this country. The Clade A lineage from Brazil was found north of this boundary, where it is widely distributed between Bragança, Pará (PA), and Guarapari, Espírito Santo (ES). In turn, members of Clade B were split into two deep reciprocally monophyletic lineages, one distributed exclusively in the localities sampled in the warm temperate southwestern Atlantic marine province (i.e., Rio de Janeiro [RJ]; Florianópolis, Santa Catarina [SC]; and Uruguay [UR]), whereas the other was found further north, in the tropical southwestern Atlantic marine province, at Rio Grande do Norte (RN) and Alagoas (AL), localities where Clade A also occurs. The southern distributional limit of the Clade A lineage from Brazil, at the ES locality (~21°S) in the tropical southwestern Atlantic, and the northernmost limit of the Clade B lineage restricted to the warm temperate southwestern Atlantic marine province, at RJ (~23°S), coincide with the boundary between these two marine provinces. The rather different oceanographic conditions between the two marine provinces (Castro and Miranda 1998) may influence this distribution. Preliminary work suggests a similar pattern in this region for other marine invertebrates, including polychaetes and echinoderms (PCP, unpublished results).

The distribution limits of the main lineages of *E. braziliensis* found in Chile also appear to correspond with biogeographic divisions. Comparing the distribution of the three Chilean clades reported in Varela and Haye (2012) with the marine ecoregions proposed by Spalding et al. (2007), the southern clade occupies the Araucanian marine ecoregion, whereas its sister lineage, the center clade, is distributed north of this ecoregion, occupying mainly the Central Chile marine ecoregion. The northern clade was found only in the Humboldtian ecoregion (Varela and Haye 2012). Lineages of the coastal isopod *Ligia occidentalis* have also distributional limits at the boundary of marine biogeographic divisions. The geographic limit between the two most divergent clades of this isopod distributed between southern Oregon and the Pacific side of the Baja California Peninsula occurs at the Point Conception biogeographic boundary, which separates the cold northern and warm southern water masses of the Oregonian and Californian marine zoogeographic provinces, respectively (Eberl et al. 2013).

It appears that the southernmost portion of the distribution of *E. braziliensis* in the Atlantic, spanning ~2000 km of coastline from Rio de Janeiro (RJ; Brazil) to Punta Negra (Uruguay), harbors a single clade (green triangles) containing very little genetic diversity (maximum COI K2P = 0.64%). This pattern suggests recent range expansions/contractions at the southern distributional limit in the Atlantic, comparable to the drastic reductions in genetic divergence observed at higher latitudes in other coastal isopods (Eberl et al. 2013; Hurtado et al. 2013), which may have resulted from glacial–postglacial climatic shifts.

Another interesting phylogeographic pattern is that the Gulf of California lineage of *E. braziliensis* appears to have diverged from an ancestor located south in the Pacific. This is in contrast to what has been found for supralittoral isopods of the genus *Tylos*, for which colonization of the northeastern Pacific appears to have proceeded from the Caribbean (Hurtado et al. 2013, 2014). Paleontological studies of the Gulf of California report that most fauna‐rich sediments found in this region have affinities with Caribbean fauna (Escalona‐Alcázar et al. 2001), and marine fossils with Caribbean affinities in the Gulf of California date back to Miocene times (Smith 1991). Nonetheless, our results suggest that the Pacific coasts south of the Gulf of California also served as a source of colonizers of coastal isopods that later differentiated within this basin, which was formed ~11.6 Ma (Helenes et al. 2009).

The dynamic geological history of the Gulf of California has contributed to extensive cryptic diversification and complex phylogeographic patterns in other intertidal isopods (Hurtado et al. 2010, 2013; Liu 2013). We attempted to comprehensively study phylogeographic patterns of *E braziliensis* in this basin, as this isopod is reported from many localities around the Gulf of California (Brusca et al. 1995). We searched for *Excirolana* specimens across the Gulf of California, including most of the localities, where *E. braziliensis* has been reported, but found this isopod at only three localities in the northern Gulf portion of the Baja California Peninsula. In contrast, we found *E. mayana* at many localities throughout the Gulf of California. The few localities sampled for *E. braziliensis* preclude meaningful phylogeographic inferences for this isopod in the Gulf of California. Nonetheless, the results suggest that, similar to other intertidal isopods, large allopatric genetic divergences within *E. braziliensis* occur inside the Gulf of California. A divergence of ~9% (COI K2P) was observed between the population of San Luis Gonzaga (SLG) and the two other localities collected, San Rafael (SR) and San Francisquito (SF). San Luis Gonzaga is separated from these by ~180 km and ~210 km, respectively. Genetic differentiation between the samples from San Rafael and San Francisquito, however, is much smaller (<1%), probably as a consequence of their geographic proximity (~30 km).

Additional sampling is necessary in the Pacific coasts between Peru and central Mexico (our analyses included samples from Costa Rica and Panama) to better understand the diversification patterns of *E. braziliensis* in this region. In the Atlantic, there is a sampling gap between Panama and Yucatan Peninsula. This isopod is also reported in several Caribbean islands, although in this region we have only found specimens of *E. mayana*. Another important sampling gap occurs on the mainland between the SM (Colombia) and PA (Brazil) localities; a region that spans the mouths of the Orinoco and Amazon rivers. We searched for *Excirolana* specimens in sandy beaches close to the Amazon mouth, but did not find any. It is possible that the freshwater discharge of the Amazon and/or the Orinoco rivers acts as an effective dispersal barrier or biogeographic filter for *E. braziliensis*. Indeed, the Amazon River constitutes an important barrier for reef fishes, as well as the boundary between the Brazilian and Caribbean biogeographic provinces (Floeter et al. 2008). Given that the holotype was collected by dredge at 37 m depth (Richardson 1912), future sampling efforts at these depths might reveal unknown diversity (e.g., speciation by depth; Goffredi et al. 2003; Ingram 2011).

The high levels of cryptic diversity detected within *E. braziliensis*, in combination with its vulnerability to anthropogenic and natural stressors that affect sandy beaches (Mclachlan and Brown 2006), bring about challenges for its preservation, requiring local‐ and regional‐level efforts for the conservation of the multiple divergent lineages. Urbanization and beach use by bathers have negatively impacted populations of *E. braziliensis* in the Rio de Janeiro area, where it is effectively absent from historically popular beaches (e.g., Copacabana and Ipanema), or it exhibits drastic changes in density and shifts in life‐history parameters (Veloso et al. 2006, 2011; Vieira et al. 2012). Human trampling and habitat modification appear to directly affect these populations (Veloso et al. 2011). Drastic reductions of *E. braziliensis* were reported in a Chilean beach following disposal of copper mine tailings (Castilla 1983). Natural impacts can also eliminate and/or cause drastic changes in the abundance of local *E. braziliensis* populations, as documented after the 2010 earthquake along the Chilean coast (Jaramillo et al. 2012). Sandy beach communities, whose intertidal fauna can be mostly (>50%) composed of direct developing, and thus dispersal‐limited, invertebrates (Grantham et al. 2003), have been largely overlooked by conservation efforts (Peterson and Bishop 2005). It has been suggested that *E. braziliensis* can be a good indicator of human impacts on sandy beach ecosystems (Veloso et al. 2011; Vieira et al. 2012). Therefore, efforts aimed at monitoring and protecting this direct‐developing and dispersal‐limited isopod may benefit other intertidal invertebrates associated with sandy shores, which usually are poorly known.

## Conflict of Interest

None declared.

## Supporting information


**Figure S1**. Majority‐rule (60%) consensus tree (RaxML bootstrap) based on the Cyt b gene (Dataset S2). Multiple individuals were examined per several localities.

**Figure S2.** Majority‐rule consensus tree (RaxML bootstrap) based on 12S rDNA gene (Dataset S1).

**Figure S3.** Majority‐rule consensus tree (RaxML bootstrap) based on 16S rDNA gene (Dataset S3).

**Figure S4.** RaxML bootstrap majority rule consensus tree of *Excirolana braziliensis*.

**Table S1.** Localities Sampled and GenBank Accession Nos. Locality ID corresponds to abbreviated name used in figures.

**Table S2.** Description of characters and substitution models for the concatenated mitochondrial dataset used to generate Figure 2 (i.e., Dataset S5).
**Table S3.** Models, parameters, and priors used in the Maximum Likelihood and Bayesian phylogenetic analyses of the concatenated mitochondrial dataset used to generate Figure 2 (i.e., Dataset S5).

**Table S4.** Description of characters and the best substitution models identified for the concatenated dataset that included the farthest outgroup taxa (Supporting Information Fig. S4 and Dataset S4).
**Table S5.** Models, parameters, and priors used in the Maximum Likelihood and Bayesian phylogenetic analyses of the for the concatenated dataset that included the farthest outgroup taxa (Supporting Information Fig. S4 and Dataset S4).

**Table S6.** Description of characters and substitution models for the analyses of the 16S rDNA dataset with only *Excirolana mayana* as outgroup (Supporting Information Fig. S3).
**Table S7.** Models, parameters, and priors used in the Maximum Likelihood and Bayesian phylogenetic analyses 16S rDNA dataset with only *Excirolana mayana* as outgroup (Supporting Information Fig. S3).

**Table S8.** Ranges of percent Kimura‐2‐parameter distances for the 12S rDNA gene among the main *Excirolana braziliensis* clades.

**Table S9.** Pairwise Kimura‐2‐parameter distances for the 16S rDNA gene. Clade to which each sample belongs is indicated.

**Table S10.** Ranges of Kimura‐2‐parameter distances for the COI gene among the main *Excirolana braziliensis* clades and the outgroup taxa *E. chiltoni* and *E. hirsuticauda*.

**Dataset S1.** Annotated 12S rDNA alignment in Nexus format.

**Dataset S2.** Annotated Cytochrome b alignment in Nexus format.

**Dataset S3.** Annotated 16S rDNA alignment in Nexus format.

**Dataset S4.** Annotated alignment alignment in Nexus format of the four concatenated genes including the farthest outgroups. Included and excluded positions are annotated.

**Dataset S5.** Annotated alignment alignment in Nexus format of the four concatenated genes excluding the farthest outgroups.
