# Supplementary figures and images for: Multiple transisthmian divergences, extensive cryptic diversity, occasional long‐distance dispersal, and biogeographic patterns in a marine coastal isopod with an amphi‐American distribution
Source: Ecol Evol. 2016 Oct 6;6(21):7794–808. doi: 10.1002/ece3.2397 (PMC6093162; doi:10.1002/ece3.2397)

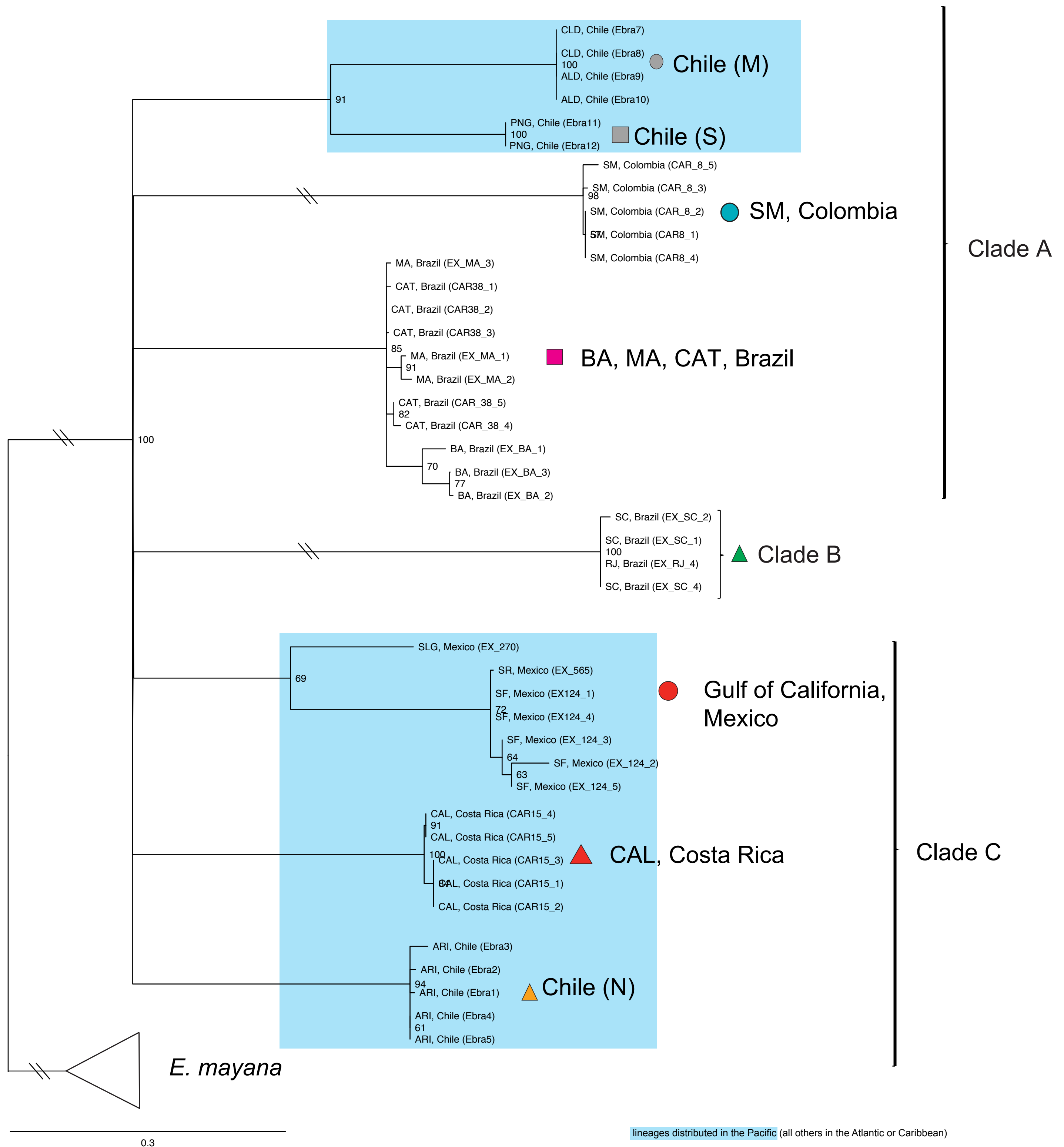

Supplement: Supplementary file 1 — Figure S1. Majority‐rule (60%) consensus tree (RaxML bootstrap) based on the Cyt b gene (Dataset S2). Multiple individuals were examined per several localities. [file ECE3-6-7794-s001.pdf]

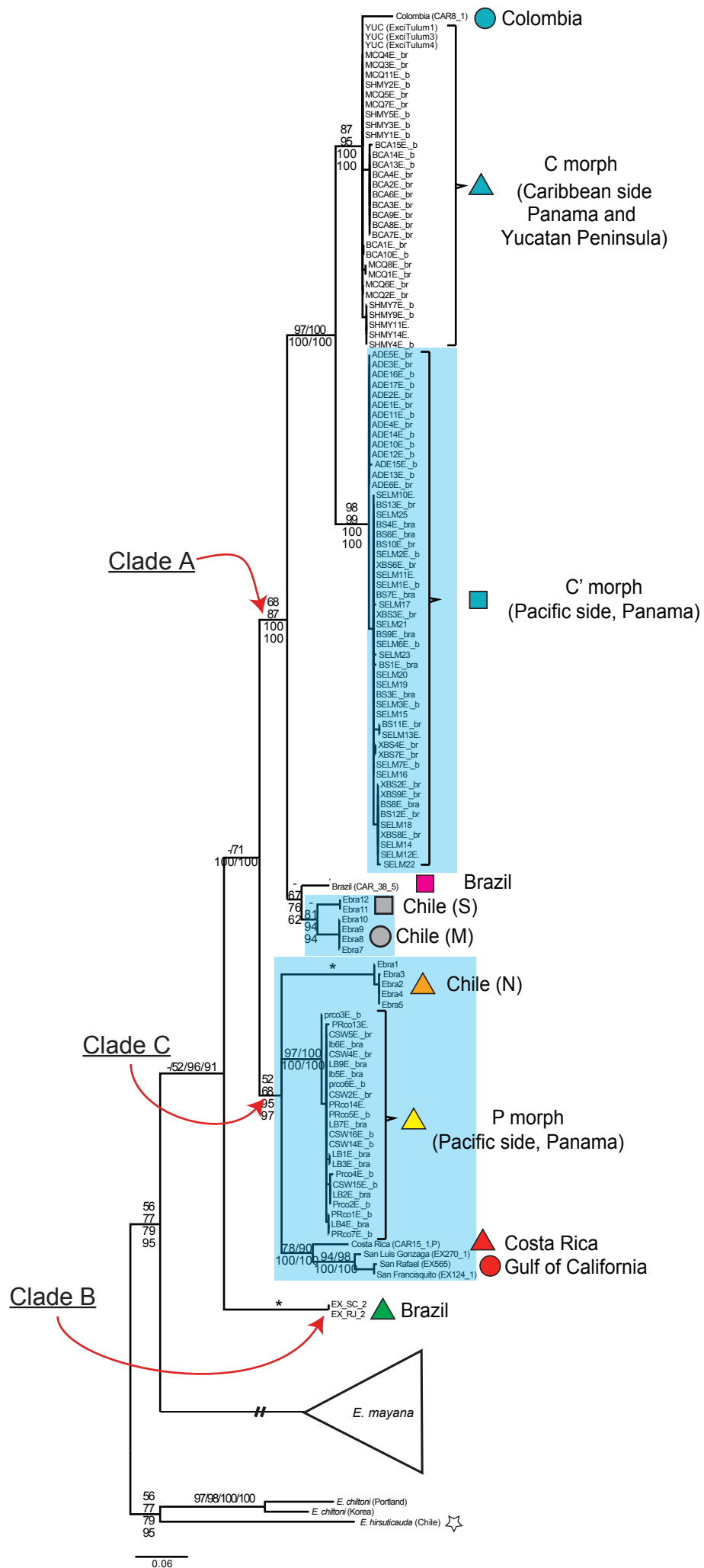

lineages distributed in the Pacific (all others in the Atlantic or Caribbean)

Supplement: Supplementary file 2 — Figure S2. Majority‐rule consensus tree (RaxML bootstrap) based on 12S rDNA gene (Dataset S1). [file ECE3-6-7794-s002.pdf]

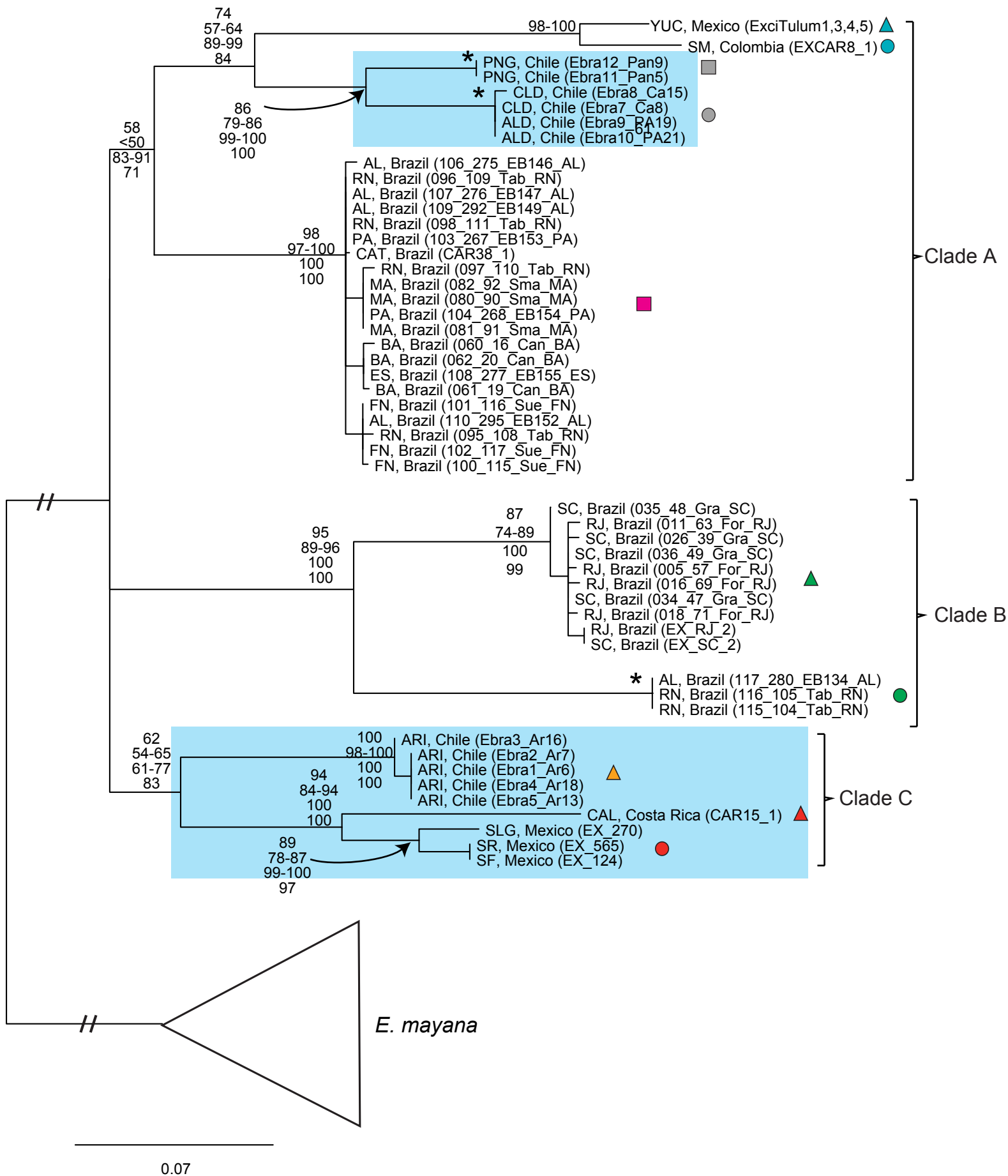

lineages distributed in the Pacific (all others in the Atlantic or Caribbean)

Supplement: Supplementary file 3 — Figure S3. Majority‐rule consensus tree (RaxML bootstrap) based on 16S rDNA gene (Dataset S3). [file ECE3-6-7794-s003.pdf]
