# Supplementary material for: Multiple transisthmian divergences, extensive cryptic diversity, occasional long‐distance dispersal, and biogeographic patterns in a marine coastal isopod with an amphi‐American distribution
Source: Ecol Evol. 2016 Oct 6;6(21):7794–808. doi: 10.1002/ece3.2397 (PMC6093162; doi:10.1002/ece3.2397)
Supplement: Supplementary file 4 — Figure S4. RaxML bootstrap majority rule consensus tree of Excirolana braziliensis. [file ECE3-6-7794-s004.pdf]

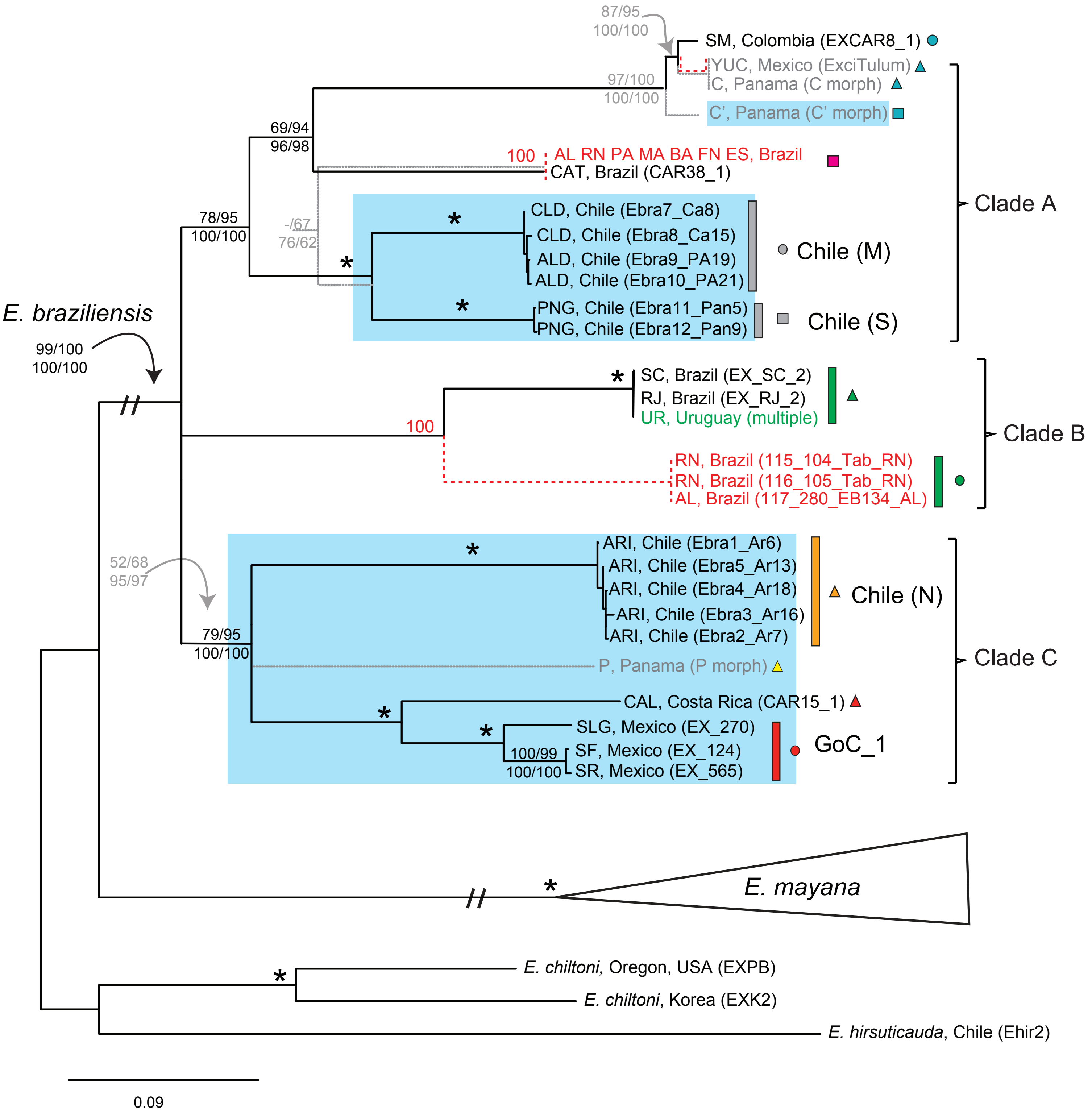

Grey = inferred from 12S rDNA gene only (branch lengths not to scale)

Red = inferred from 16S rDNA only (branch lengths not to scale)

lineages distributed in the Pacific (all others in the Atlantic or Caribbean)

Green = inferred from COI only (branch lengths not to scale)
