# Supplementary material for: Multiple transisthmian divergences, extensive cryptic diversity, occasional long‐distance dispersal, and biogeographic patterns in a marine coastal isopod with an amphi‐American distribution
Source: Ecol Evol. 2016 Oct 6;6(21):7794–808. doi: 10.1002/ece3.2397 (PMC6093162; doi:10.1002/ece3.2397)
Supplement: Supplementary file 5 — Table S1. Localities Sampled and GenBank Accession Nos. Locality ID corresponds to abbreviated name used in figures. [file ECE3-6-7794-s005.docx]

Supporting Table S1. Localities Sampled and GenBank Accession Nos. Locality ID corresponds to abbreviated name used in figures. Newly generated sequences are Acc. Nos. KT122XXX and KX5309XX. Previously published sequences are Acc. Nos. FJ541XXX (Varela and Haye, 2012), KP184XXX (Sponer and Lessios, 2009), KT8701XX (Tourinho et al., 2016).

| **Locality ID** | **Locality Name** | **Country** | **Latitude** | **Longitude** | **Cytb** | **COI** | **12S** | **16S** |  |
| --- | --- | --- | --- | --- | --- | --- | --- | --- | --- |
| *Excirolana braziliensis* | | | | | | | | | |
| UR | Several localities (spanning ca. 240 km of coastline) | Uruguay |  |  |  |  | KT870118- KT870130 |  |  |
| AL | Jequia da Praia, Alagoas | Brazil | 10.04° S | 36.02° W |  |  |  | KT122410 |  |
|  |  |  |  |  |  |  |  | KT122411 |  |
|  |  |  |  |  |  |  |  | KT122412 |  |
|  |  |  |  |  |  |  |  | KT122413 |  |
|  |  |  |  |  |  |  |  | KT122414 |  |
| BA | Canavieiras Beach, Bahia | Brazil | 15.66° S | 38.94° W | KT122698 |  |  | KT122424 |  |
|  |  |  |  |  | KT122699 |  |  | KT122425 |  |
|  |  |  |  |  | KT122700 |  |  | KT122426 |  |
| CAT | Catuama Beach | Brazil |  |  | KT122593 | KT122546 | KT122499 | KT122433 |  |
|  |  |  |  |  | KT122594 |  |  |  |  |
|  |  |  |  |  | KT122612 |  |  |  |  |
|  |  |  |  |  | KT122613 |  |  |  |  |
|  |  |  |  |  | KT122614 |  |  |  |  |
| ES | Prainha Beach, Guarapari, Espirito Santos | Brazil | 20.66° S | 40.5° W |  |  |  | KT122442 |  |
| FN | Sueste Beach, Fernando de Noronha Archipelago, Pernambuco | Brazil | 3.87° S | 32.42° W |  |  |  | KT122444 |  |
|  |  |  |  |  |  |  |  | KT122445 |  |
|  |  |  |  |  |  |  |  | KT122446 |  |
| MA | Sao Marcos Beach, Sao Luis, Maranhao | Brazil | 2.49° S | 44.29° W | KT122716 |  |  | KT122457 |  |
|  |  |  |  |  | KT122717 |  |  | KT122458 |  |
|  |  |  |  |  | KT122718 |  |  | KT122459 |  |
| PA | Ajuruteua Beach, Braganza, Para | Brazil | 0.84° S | 46.6° W |  |  |  | KT122462 |  |
|  |  |  |  |  |  |  |  | KT122463 |  |
| RJ | Fora Beach Rio de Janeiro | Brazil | 22.95° S | 43.16° W | KT122719 | KT122547 | KT122523 | KT122471 |  |
|  |  |  |  |  |  |  |  | KT122472 |  |
|  |  |  |  |  |  |  |  | KT122473 |  |
|  |  |  |  |  |  |  |  | KT122474 |  |
|  |  |  |  |  |  |  |  | KT122475 |  |
| RN | Barra de Tabatinga, Rio Grande do Norte | Brazil | 6.06° S | 35.1° W |  |  |  | KT122476 |  |
|  |  |  |  |  |  |  |  | KT122477 |  |
|  |  |  |  |  |  |  |  | KT122478 |  |
|  |  |  |  |  |  |  |  | KT122479 |  |
|  |  |  |  |  |  |  |  | KT122480 |  |
|  |  |  |  |  |  |  |  | KT122481 |  |
| SC | Sao Francisco do Sul Santa Catarina | Brazil | 26.24° S | 48.5° W |  |  |  | KT122484 |  |
|  |  |  |  |  |  |  |  | KT122485 |  |
|  |  |  |  |  |  |  |  | KT122486 |  |
|  |  |  |  |  |  |  |  | KT122487 |  |
|  |  |  |  |  | KT122720 |  |  |  |  |
|  |  |  |  |  | KT122721 | KT122548 | KT122524 | KT122488 |  |
|  |  |  |  |  | KT122722 |  |  |  |  |
| ALD | Puerto Aldea | Chile | 30.3° S | 71.2° W | KT122624 |  | KT122510 | KT122415 |  |
|  |  |  |  |  | KT122633 | FJ541222 |  | KT122416 |  |
|  |  |  |  |  |  | FJ541223 |  |  |  |
| ARI | Arica | Chile | 18.4° S | 70.3° W | KT122623 |  | KT122506 | KT122419 |  |
|  |  |  |  |  | KT122627 |  | KT122507 | KT122420 |  |
|  |  |  |  |  | KT122628 | KT122544 | KT122508 | KT122421 |  |
|  |  |  |  |  | KT122629 |  | KT122509 | KT122422 |  |
|  |  |  |  |  | KT122630 | FJ541198- FJ541201 |  | KT122423 |  |
|  |  |  |  |  |  | FJ541203 |  |  |  |
| CLD | Caldera | Chile | 27° S | 70.8° W | KT122631 | FJ541215 |  | KT122435 |  |
|  |  |  |  |  | KT122632 | FJ541217 |  | KT122436 |  |
| PNG | Playa Pangua | Chile | 34.5° S | 72° W | KT122625 | FJ541229 |  | KT122465 |  |
|  |  |  |  |  | KT122626 | FJ541230 |  | KT122466 |  |
| SM | Santa Marta | Colombia |  |  | KT122595 |  |  |  |  |
|  |  |  |  |  | KT122596 |  |  |  |  |
|  |  |  |  |  | KT122597 |  |  |  |  |
|  |  |  |  |  | KT122616 | KT122551 | KT122540 | KT122492 |  |
|  |  |  |  |  | KT122617 |  |  |  |  |
| CAL | Caldera | Costa Rica |  |  | KT122603 | KT122545 | KT122502 | KT122432 |  |
|  |  |  |  |  | KT122604 |  |  |  |  |
|  |  |  |  |  | KT122605 |  |  |  |  |
|  |  |  |  |  | KT122606 |  |  |  |  |
|  |  |  |  |  | KT122607 |  |  |  |  |
| SF | San Francisquito | Mexico |  |  | KT122639 | KT122549 | KT122526 | KT122489 |  |
|  |  |  |  |  | KT122640 |  |  |  |  |
|  |  |  |  |  | KT122641 |  |  |  |  |
|  |  |  |  |  | KT122726 |  |  |  |  |
| SLG | San Luis Gonzaga | Mexico |  |  | KT122643 | KT122550 | KT122528 | KT122491 |  |
| SR | San Rafael | Mexico |  |  | KT122678 | KT122552 | KT122543 | KT122493 |  |
| C morph | Several localities (Caribbean) | Panama |  |  |  |  | KP184594-KP184598 |  |  |
|  |  |  |  |  |  |  | KP184600-KP184603 |  |  |
|  |  |  |  |  |  |  | KP184606-KP184626 |  |  |
| YUC | near Tulum, Quintana Roo | Mexico |  |  |  |  | KX530938-KX530940 | KX530933-KX530936 |  |
| C’ morph | Several Localities (Pacific) | Panama |  |  |  |  | KP184650-KP184701 |  |  |
| P morph | Several Localities (Pacific) | Panama |  |  |  |  | KP184627-KP184649 |  |  |
| *Excirolana chiltoni* (outgroup) | | | | | | | | | |
|  | Korea | Korea |  |  | KT122765 | KT122553 | KT122541 | KT122439 |  |
|  | Cape Lookout, Oregon | USA |  |  | KT122766 | KT122554 | KT122542 | KT122440 |  |
| *Excirolana hirsuticauda* (outgroup) | | | | | | | | | |
| TRN | Tranaquepe | Chile | 38.17°S | 73.52 °W | KT122764 | KT122555 | KT122512 | KT122441 |  |

**References**

SPONER, R. & LESSIOS, H. A. 2009. Mitochondrial phylogeography of the intertidal Isopod *Excirolana braziliensis* on the two sides of the Isthmus of Panama. *Smithsonian Contributions to the Marine Sciences,* 38**,** 219-228.

TOURINHO, J. L., MÁRQUEZ, A., CELENTANO, E. & DEFEO, O. 2016. A new evolutionary unit in the *Excirolana braziliensis* species complex. *Brazilian Journal of Oceanography,* 64(2)**,** 197-202.

VARELA, A. I. & HAYE, P. A. 2012. The marine brooder *Excirolana braziliensis* (Crustacea: Isopoda) is also a complex of cryptic species on the coast of Chile. *Revista Chilena de Historia Natural,* 85**,** 495-502. doi: 10.4067/S0716-078X2012000400011.
