# Supplementary material for: Multiple transisthmian divergences, extensive cryptic diversity, occasional long‐distance dispersal, and biogeographic patterns in a marine coastal isopod with an amphi‐American distribution
Source: Ecol Evol. 2016 Oct 6;6(21):7794–808. doi: 10.1002/ece3.2397 (PMC6093162; doi:10.1002/ece3.2397)
Supplement: Supplementary file 6 — Table S2. Description of characters and substitution models for the concatenated mitochondrial dataset used to generate Figure 2 (i.e., Dataset S5). Table S3. Models, parameters, and priors used in the Maximum Likelihood and Bayesian phylogenetic analyses of the concatenated mitochondrial dataset used to generate Figure 2 (i.e., Dataset S5). [file ECE3-6-7794-s006.docx]

**Table S2. Description of characters and substitution models for the concatenated mitochondrial dataset used to generate Fig. 2 (i.e., Dataset S5).**

Number of characters per gene region that were excluded from and included in the phylogenetic analyses. The number of parsimony informative characters is based on included characters only. Best model selected by jModelTest according to each criterion (AIC, AICc, BIC) and its corresponding weight.

| Gene | Samples | Total characters ^a^ | Excluded characters ^b^ | Included characters | Parsimony informative | AICc (weight) | AIC (weight) | BIC (weight) |
| --- | --- | --- | --- | --- | --- | --- | --- | --- |
| 16S rDNA | 54 | 455 | 164 | 291 | 106 | TVM+I+G (0.76) | TVM+I+G (0.61) | TVM+I+G (0.70) |
| 12S rDNA | 54 | 485 | 117 | 368 | 119 | GTR+I+G (1.00) | GTR+I+G (1.00) | GTR+I+G (1.00) |
| Cytb | 54 | 309 | 0 | 309 | 153 | TIM2+I+G (1.00) | TIM2+I+G (1.00) | TIM2+I+G (1.00) |
| COI | 54 | 543 | 0 | 543 | 213 | TIM1+I+G (0.86) | TIM1+I+G (0.75) | TIM1+I+G (0.86) |
| MT | 54 | 1792 | 281 | 1511 | 591 | GTR+I+G (0.99) | GTR+I+G (0.99) | GTR+I+G (0.85) |

^a^ Total number of characters in the alignment, including gaps

**^b^** List of excluded characters is provided in Supporting Dataset files

MT = combined mitochondrial genes

**Table S3. Models, parameters, and priors used in the Maximum Likelihood and Bayesian phylogenetic analyses of the concatenated mitochondrial dataset used to generate Fig. 2 (i.e., Dataset S5).**

| Method | Model and Priors^1^ | Partitioning scheme^2^ | iterations generations/bootstrap replicates | Sample frequency | runs/ chains | burnin | ASDSF^3^ | Bayes Factors^4^ /ML scores (-lLn) | ESS^4,5^  > 200 | PSRF^6^ |
| --- | --- | --- | --- | --- | --- | --- | --- | --- | --- | --- |
| RaxML | GTR G | 1 | 1000 | na | na | na | na | -14933.987 | na | na |
| Garli | GTR G | 1 | 1000 | na | na | na | na | -14623.491 | na | na |
| MrBayes | GTR G | 1 | 10,000,000 | 1,000 | 4/4 | 25% | 0.0055749 | -14505367 | yes | 1 |
| MrBayes | GTR G | 5(12S+16s+Cytb1,Cytb2+COI2,Cytb3,COI1,COI3)^7^ | 10,000,000 | 1,000 | 4/4 | 25% | 0.0044567 | -13790.593 | yes | 1 |
| Phycas | polytomy prior | 1 | 500,000 | 100 | na | 20% | na | -14570.662 | na | na |
|  |  |  |  |  |  |  |  |  |  |  |
|  |  |  |  |  |  |  |  |  |  |  |

^1^ All others default; ^2^ different partitions separated by comma; ^3^ Average standard deviation of split frequencies; ^4^ estimated in Tracer v.1.5;

^5^ Effective Sample Size; ^6^ Potential Scale Reduction Factor for all parameters; ^7^ Partition finder 1.0 (SYM+I+G; TrN+I; TrN+G; TrN+G; GTR+G)
