# Supplementary material for: Multiple transisthmian divergences, extensive cryptic diversity, occasional long‐distance dispersal, and biogeographic patterns in a marine coastal isopod with an amphi‐American distribution
Source: Ecol Evol. 2016 Oct 6;6(21):7794–808. doi: 10.1002/ece3.2397 (PMC6093162; doi:10.1002/ece3.2397)
Supplement: Supplementary file 7 — Table S4. Description of characters and the best substitution models identified for the concatenated dataset that included the farthest outgroup taxa (Supporting Information Fig. S4 and Dataset S4). Table S5. Models, parameters, and priors used in the Maximum Likelihood and Bayesian phylogenetic analyses of the for the concatenated dataset that included the farthest outgroup taxa (Supporting Information Fig. S4 and Dataset S4). [file ECE3-6-7794-s007.docx]

Table S4. Description of characters and the best substitution models identified for the concatenated dataset that included the farthest outgroup taxa (Supporting Information Fig. S4 and Dataset S4). Number of characters per gene region that were excluded from and included in the phylogenetic analyses. The number of parsimony informative characters is based on included characters only. Best model selected by jModelTest according to each criterion (AIC, AICc, BIC) and its corresponding weight.

| Gene | Samples | Total characters ^a^ | Excluded characters ^ab^ | Included characters | Parsimony informative | AICc (weight) | AIC  (weight) | BIC (weight) |
| --- | --- | --- | --- | --- | --- | --- | --- | --- |
| 16S rDNA | 57 | 453 | 165 | 288 | 104 | TVMef+I+G (0.87) | TVMef+I+G (0.53) | TVMef+I  +G  (0.87) |
| 12S rDNA | 57 | 486 | 114 | 372 | 130 | GTR+I+G (0.81) | GTR+I+G (0.97) | SYM+I+G (0.44) |
| Cytb | 57 | 309 | 0 | 309 | 155 | TIM3+I+G (0.65) | TIM3+I+G (0.79) | TIM3+I+G  (0.57) |
| COI | 57 | 543 | 0 | 543 | 215 | GTR+I+G (0.76) | GTR+I+G (0.92) | TIM2+I+G (0.58) |
| MT | 57 | 1791 | 279 | 1512 | 604 | GTR+I+G (0.99) | GTR+I+G (0.99) | SYM +I+G (0.55) |

^a^ Total number of characters in the alignment, including gaps

**^b^** Criteria for character exclusion are described in a nexus file in the supporting information

MT = combined mitochondrial genes

Table S5. Models, parameters, and priors used in the Maximum Likelihood and Bayesian phylogenetic analyses of the for the concatenated dataset that included the farthest outgroup taxa (Supporting Information Fig. S4 and Dataset S4).

| Method | Model and Priors^1^ | Partitioning scheme^2^ | iterations generations/bootstrap replicates | Sample frequency | runs/ chains | burnin | ASDSF^3^ | Bayes Factors^4^ /ML scores (-lLn) | ESS^4,5^  > 200 | PSRF^6^ |
| --- | --- | --- | --- | --- | --- | --- | --- | --- | --- | --- |
| RaxML | GTR G | 1 | 1000 | na | na | na | na | -16475.625 | na | na |
| Garli | GTR G | 1 | 1000 | na | na | na | na | -15595.338 | na | na |
| MrBayes | GTR G | 1 | 10,000,000 | 1,000 | 4/4 | 25% | 0.0057009 | -16258.905 | yes | 1 |
| MrBayes | GTR G | 5(12S+16s+Cytb1,Cytb2+COI2,Cytb3,COI1,COI3)^7^ | 10,000,000 | 1,000 | 4/4 | 25% | 0.0042792 | -15331.528 | yes | 1 |
| Phycas | polytomy prior | 1 | 500,000 | 100 | na | 20% | na | -16341.630 | na | na |
|  |  |  |  |  |  |  |  |  |  |  |
|  |  |  |  |  |  |  |  |  |  |  |

^1^ All others default; ^2^ different partitions separated by comma; ^3^ Average standard deviation of split frequencies; ^4^ estimated in Tracer v.1.5;

^5^ Effective Sample Size; ^6^ Potential Scale Reduction Factor for all parameters; ^7^ Partition finder 1.0 (SYM+I+G; TrN+I; TrN+G; TrN+G; GTR+G)
