# Supplementary material for: Multiple transisthmian divergences, extensive cryptic diversity, occasional long‐distance dispersal, and biogeographic patterns in a marine coastal isopod with an amphi‐American distribution
Source: Ecol Evol. 2016 Oct 6;6(21):7794–808. doi: 10.1002/ece3.2397 (PMC6093162; doi:10.1002/ece3.2397)
Supplement: Supplementary file 8 — Table S6. Description of characters and substitution models for the analyses of the 16S rDNA dataset with only Excirolana mayana as outgroup (Supporting Information Fig. S3). Table S7. Models, parameters, and priors used in the Maximum Likelihood and Bayesian phylogenetic analyses 16S rDNA dataset with only Excirolana mayana as outgroup (Supporting Information Fig. S3). [file ECE3-6-7794-s008.docx]

Table S6. Description of characters and substitution models for the analyses of the 16S rDNA dataset with only *E. mayana* as outgroup (Supporting Information Fig. S3). Number of characters per that were excluded from and included in the phylogenetic analyses. The number of parsimony informative characters is based on included characters only. Best model selected by jModelTest according to each criterion (AIC, AICc, BIC) and its corresponding weight.

| Gene | Samples | Total characters ^a^ | Excluded characters ^ab^ | Included characters | Parsimony informative | AIC (weight) | AICc  (weight) | BIC (weight) |
| --- | --- | --- | --- | --- | --- | --- | --- | --- |
| 16S rDNA | 86 | 515 | 220 | 295 | 119 | SYM+I+G (0.35) | TPM2+G (0.60) | TPM2+I+G (0.64) |

^a^ Total number of characters in the alignment, including gaps.

**^b^** Criteria for character exclusion are described in a nexus file in the supporting information.

MT = combined mitochondrial genes

Table S7. Models, parameters, and priors used in the Maximum Likelihood and Bayesian phylogenetic analyses 16S rDNA dataset with only *E. mayana* as outgroup (Supporting Information Fig. S3).

| Method | Model and Priors^1^ | Partitioning scheme^2^ | iterations generations/bootstrap replicates | Sample frequency | runs/ chains | burnin | ASDSF^3^ | Bayes Factors^4^ /ML scores (-lLn) | ESS^4,5^  > 200 | PSRF^6^ |
| --- | --- | --- | --- | --- | --- | --- | --- | --- | --- | --- |
| RaxML | GTR G | 1 | 1000 | na | na | na | na | -2411.56 | na | na |
| Garli | GTR G | 1 | 1000 | na | na | na | na | -2403.67 | na | na |
| Garli | SYM I G | 1 | 1000 | na | na | na | na | -2405.35 | na | na |
| Garli | TPM2 I G | 1 | 1000 | na | na | na | na | -2410.15 | na | na |
| Garli | TPM2G | 1 | 1000 | na | na | na | na | -2416.25 | na | na |
| MrBayes | GTR G | 1 | 10,000,000 | 1,000 | 4/4 | 25% | 0.01 | -2662.85 | yes | 1 |
| MrBayes | GTR G I | 1 | 10,000,000 | 1,000 | 4/4 | 25% | 0.01 | -2646.09 | yes | 1 |
| MrBayes | SYM I G | 1 | 10,000,000 | 1,000 | 4/4 | 25% | 0.01 | -2811.34 | yes | 1 |
| Phycas | GTR G; polytomy prior | 1 | 500,000 | 100 | na | 20% | na | -2554.17 | yes | na |
|  |  |  |  |  |  |  |  |  |  |  |
|  |  |  |  |  |  |  |  |  |  |  |

^1^ All others default; ^2^ different partitions separated by comma; ^3^ Average standard deviation of split frequencies; ^4^ estimated in Tracer v.1.5; ^5^ Effective Sample Size; ^6^ Potential Scale Reduction Factor for all parameters
