# Supplementary material for: Multiple transisthmian divergences, extensive cryptic diversity, occasional long‐distance dispersal, and biogeographic patterns in a marine coastal isopod with an amphi‐American distribution
Source: Ecol Evol. 2016 Oct 6;6(21):7794–808. doi: 10.1002/ece3.2397 (PMC6093162; doi:10.1002/ece3.2397)
Supplement: Supplementary file 9 — Table S8. Ranges of percent Kimura‐2‐parameter distances for the 12S rDNA gene among the main Excirolana braziliensis clades. [file ECE3-6-7794-s009.docx]

Table S8. Ranges of percent Kimura-2-parameter distances for the 12S rDNA gene among the main *Excirolana braziliensis* clades. Values on diagonal show maximum within-clade divergence

|  |  | Clade A | | | | | | Clade C | | | | Clade B |
| --- | --- | --- | --- | --- | --- | --- | --- | --- | --- | --- | --- | --- |
|  |  | Colombia | Panama C morph | Panama C' morph | Brazil | Chile (S) | Chile (M) | Chile (N) | Panama P morph | Costa Rica (Pacific) | Mexico (Pacific) | SC/RJ Brazil |
| Clade A | Colombia | NA |  |  |  |  |  |  |  |  |  |  |
|  | Panama C morph | 3.9-4.8 | 1.2 |  |  |  |  |  |  |  |  |  |
|  | Panama C' morph | 7.8-9.1 | 4.8-7.3 | 1.5 |  |  |  |  |  |  |  |  |
|  | Brazil | 10.0 | 6.8-7.8 | 7.7-9.1 | NA |  |  |  |  |  |  |  |
|  | Chile (S) | 10.8 | 7.7.-8.7 | 8.2-9.5 | 5.1 | 0 |  |  |  |  |  |  |
|  | Chile (M) | 11.4 | 8.6-9.7 | 10.0-11.4 | 6.4 | 3.9 | 0 |  |  |  |  |  |
| Clade C | Chile (N) | 16.0-16.5 | 13.9-15.9 | 15.0-17.0 | 15.0-15.5 | 12.6-13.0 | 10.8-11.2 | 0.4 |  |  |  |  |
|  | Panama P morph | 11.7-12.7 | 9.9-11.8 | 11.7-14.1 | 9.0-9.9 | 8.1-8.9 | 7.2-8.1 | 10.3-11.6 | 1.2 |  |  |  |
|  | Costa Rica (Pacific) | 12.6 | 11.1-12.5 | 10.7-12.0 | 13.0 | 11.1 | 11.2 | 12.9-13.4 | 7.7-8.5 | NA |  |  |
|  | Mexico (Pacific) | 10.3-11.7 | 9.4-11.2 | 9.8-11.2 | 9.4-11.2 | 9.8-11.6 | 12.6-13.5 | 12.1-13.0 | 8.5-10.8 | 7.7-8.5 | 1.5 |  |
| Clade B | SC/RJ Brazil | 14.4 | 12.9-13.9 | 14.4-16.4 | 15.4 | 15.0 | 15.5 | 14.9-15.4 | 13.6-15.5 | 13.5 | 13.9-14.9 | 0 |
