# Supplementary material for: Multiple transisthmian divergences, extensive cryptic diversity, occasional long‐distance dispersal, and biogeographic patterns in a marine coastal isopod with an amphi‐American distribution
Source: Ecol Evol. 2016 Oct 6;6(21):7794–808. doi: 10.1002/ece3.2397 (PMC6093162; doi:10.1002/ece3.2397)
Supplement: Supplementary file 11 — Table S10. Ranges of Kimura‐2‐parameter distances for the COI gene among the main Excirolana braziliensis clades and the outgroup taxa E. chiltoni and E. hirsuticauda. [file ECE3-6-7794-s011.docx]

Table S10. Ranges of Kimura-2-parameter distances for the COI gene among the main *Excirolana braziliensis* clades and the outgroup taxa *E. chiltoni* and *E. hirsuticauda*. Values on diagonal show maximum within-clade divergence.

|  |  | Clade A | | | | Clade B | Clade C | | | Outgroup | |
| --- | --- | --- | --- | --- | --- | --- | --- | --- | --- | --- | --- |
|  |  | Brazil | Chile (M) | Chile (S) | Colombia | Brazil | Chile (N) | Costa Rica | GOC | *E. chiltoni* | *E. hirsuticauda* |
| Clade A | Brazil | n/a |  |  |  |  |  |  |  |  |  |
|  | Chile (M) | 0.2047 | 0.0111 |  |  |  |  |  |  |  |  |
|  | Chile (S) | 0.1945–0.2047 | 0.1518–0.164 | 0.0056 |  |  |  |  |  |  |  |
|  | Colombia | 0.1912–0.1937 | 0.1773–0.1877 | 0.2049–0.2076 | n/a |  |  |  |  |  |  |
| Clade B | Brazil | 0.179 | 0.2138–0.2266 | 0.2228–0.2304 | 0.2133–0.216 | 0.0064 |  |  |  |  |  |
| Clade C | Chile (N) | 0.2013–0.2023 | 0.2159–0.2369 | 0.2362–0.2464 | 0.2315–0.234 | 0.1969–0.2027 | 0.0112 |  |  |  |  |
|  | Costa Rica | 0.2228–0.233 | 0.1996–0.2073 | 0.2075–0.2127 | 0.2066 | 0.2133–0.2142 | 0.1941–0.2039 | n/a |  |  |  |
|  | GOC | 0.2039 | 0.1976–0.2162 | 0.1896–0.2047 | 0.1848–0.1924 | 0.2115–0.2201 | 0.1917–0.2101 | 0.1613–0.168 | 0.0923 |  |  |
| Outgroup | *E. chiltoni* | 0.213–0.2293 | 0.1915–0.273 | 0.1824–0.2611 | 0.2211–0.289 | 0.229–0.2622 | 0.1896–0.2305 | 0.2125–0.2425 | 0.2102–0.2379 | 0.1845 |  |
|  | *E. hirsuticauda* | 0.1976–0.2361 | 0.2497–0.2581 | 0.2667–0.2725 | 0.2302 | 0.2472–0.2494 | 0.234–0.2442 | 0.2222 | 0.2536–0.2796 | 0.2125–0.2529 | n/a |
