# Supplementary material for: Multiple transisthmian divergences, extensive cryptic diversity, occasional long‐distance dispersal, and biogeographic patterns in a marine coastal isopod with an amphi‐American distribution
Source: Ecol Evol. 2016 Oct 6;6(21):7794–808. doi: 10.1002/ece3.2397 (PMC6093162; doi:10.1002/ece3.2397)
Supplement: Supplementary file 17 [file ECE3-6-7794-s017.docx]

**Supporting Dataset Descriptions**

Dataset S1. Annotated 12S rDNA alignment in Nexus format.

Dataset S2. Annotated Cytochrome b alignment in Nexus format.

Dataset S3. Annotated 16S rDNA alignment in Nexus format.

Dataset S4. Annotated alignment alignment in Nexus format of the four concatenated genes including the farthest outgroups. Included and excluded positions are annotated.

Dataset S5. Annotated alignment alignment in Nexus format of the four concatenated genes excluding the farthest outgroups.
