# Supplementary material for: Multiple transisthmian divergences, extensive cryptic diversity, occasional long‐distance dispersal, and biogeographic patterns in a marine coastal isopod with an amphi‐American distribution
Source: Ecol Evol. 2016 Oct 6;6(21):7794–808. doi: 10.1002/ece3.2397 (PMC6093162; doi:10.1002/ece3.2397)
Supplement: Supplementary file 18 [file ECE3-6-7794-s018.docx]

**Supporting Figure legends**

**Figure S1**. Majority-rule (60%) consensus tree (RaxML bootstrap) based on the Cyt b gene (Dataset S2). Multiple individuals were examined per several localities.  Numbers by branches are bootstrap support.  Tree is rooted at branch joining *E. mayana* and *E. braziliensis*. Clades A, B, and C were labeled according to the 12S rDNA analyses (see text).  This split received 100% support. Colors and shapes correspond to lineages, clades, or localities in other figures.  Blue-shading indicates taxa found in the Pacific Ocean.

**Figure S2.** Majority-rule consensus tree (RaxML bootstrap) based on 12S rDNA gene (Dataset S1). Numbers by nodes indicate the corresponding Bootstrap Support (BS; top or left) for Maximum likelihood (RaxML and Garli, respectively); and Posterior Probabilities (PP; bottom or right) for Bayesian inference methods (MrBayes and Phycas, respectively). * denotes nodes that received 100% support for all methods. -- denotes nodes receiving < 50% support for the corresponding method. Nodes receiving < 50% support for all methods were collapsed. Colors and shapes correspond to lineages, clades, or localities in other figures. Blue-shading indicates taxa found in the Pacific Ocean.

**Figure S3.** Majority-rule consensus tree (RaxML bootstrap) based on 16S rDNA gene (Dataset S3). Numbers by nodes indicate the corresponding Bootstrap Support (BS; top or left) for Maximum likelihood (RaxML and Garli, respectively); and Posterior Probabilities (PP; bottom or right) for Bayesian inference methods (MrBayes and Phycas, respectively). * denotes nodes that received 100% support for all methods. -- denotes nodes receiving < 50% support for the corresponding method. Nodes receiving < 50% support for all methods were collapsed. Colors and shapes correspond to lineages, clades,

**Figure S4.** RaxML bootstrap majority rule consensus tree of *Excirolana braziliensis*.

Tree was inferred from the dataset (Dataset S4) based on four concatenated mitochondrial genes (12S+16S+Cytb+COI). The farthest outgroups were used. Lineages indicated by red font and branches were re-drawn on the basis of the 16S rDNA dataset, and those indicated by grey font and branches were re-drawn on the basis of the 12S rDNA dataset. Numbers by nodes indicate the corresponding Bootstrap Support (BS; top or left) for Maximum likelihood (RaxML and Garli, respectively); and Posterior Probabilities (PP; bottom or right) for Bayesian inference methods (MrBayes and Phycas, respectively), including all partitioning schemes. * denotes nodes that received 100% support for all methods. -- denotes nodes receiving < 50% support for the corresponding method. Nodes receiving < 50% support for all methods were collapsed. Colors and shapes correspond to lineages, clades, or localities in other figures. Blue-shading indicates taxa found in the Pacific Ocean.
